# Community Engagement in an African American Community during the COVID-19 Pandemic: Assessment of a Train-the-Trainer COVID-19 Curriculum

**Published:** 2024-09-24

**Authors:** Alan Becker, John S Luque, Carlos A Reyes Ortiz, Donald Axelrad, Rima Tawk, Torhonda Lee, Jessica Saintibert, Cindy Telfort, Cynthia Harris

**Affiliations:** 1 Institute of Public Health, College of Pharmacy and Pharmaceutical Sciences, Florida A&M University, USA; 2 Department of Graduate Public Health, College of Veterinary Medicine, Tuskegee University, Tuskegee, Alabama

**Keywords:** COVID-19 pandemic, Community research, Community education, African Americans, Community engagement

## Abstract

The purpose of this study was to produce a culturally sensitive COVID-19 curriculum for primarily African Americans related to the risks of the health impacts of COVID-19. Community engagement was initiated to deliver risk communication for COVID-19 to community trainers who, in turn, recruited and trained additional community trainers. Florida Agricultural and Mechanical University Training investigators conducted training from May 2021 through January 2023. A total of twenty community trainers were trained over a two-year period for 2021 and 2023. Community trainers recruited and trained two hundred additional trainers (one hundred trainers for each year). Data were collected in Qualtrics. © and evaluated the effectiveness of the training delivered to the community trainers measuring learning gains.

The differences between pre-and post-learning gains (n=146) were positive (+451 learning gains difference; average learning gain (8.8%). The pre-post median number of correct answers improved (Wilcoxon signed-rank test, p<0.001). Eighteen questions (out of 35) had substantial differences in proportions at the α=.05 level (McNemar test). The overall train-the-trainer community training satisfaction survey had a mean of 4.7 (±0.7), indicating high satisfaction of the community trainers. Community trainers improved their knowledge of COVID-19 risks. These behavioral and preventative measures, such as social distancing, wearing face masks, adopting sanitation, adhering to quarantine-isolation practices, rejecting misinformation, and discussing the pros and cons of vaccinations and boosters, likely prevented some COVID-19 infections.

## Introduction

African Americans and other minorities in the US are at a higher risk of contracting COVID-19 than whites when adjusted for age [1]. Hispanics and African Americans received the lowest COVID-19 boosters in 2022, 15% and 8%, respectively [2]. In addition, African Americans and other minorities usually residing in vulnerable communities are under chronic stressors and are more likely to have preexisting conditions and other comorbidities than their white counterparts, which makes them more susceptible to severe complications after contracting COVID-19 [3].

## Purpose and Objectives

Having strong community support and recognizing barriers is critical [4]. Collaborating with community trainers can succeed because of their trusted relationships through action, transparency, goodwill, and shared values [5]. Community stakeholders and residents examined COVID-19 perceptions of prevention and testing in African American communities. Vaccine hesitancy is primarily driven by mistrust, fear, and lack of information [5]. including misinformation and mixed messages, suspicion of historical injustices, and distrust of government authorities.

The study objective was to provide train-the-trainer COVID-19 vaccine information, outreach, education, and training of primarily to train the African American community about prevention, transmission, and other COVID-19 health effects. FAMU secured funding from Florida Department of Health to implement the train-the-trainer study. We recruited community trainers through our community network and conducted four ZOOM training sessions for trainers to implement their recruitment to train additional community trainers. In addition, these trainers engaged community residents at large events, barber shops, churches where they answered questions related to COVID-19 and vaccines. The community had input in the training materials, and it was modified based on the community input for research questions and COVID-19 misinformation in the community and the community also contributed to the research questions.

## Intervention Approach

Misinformation and distrust may cause reluctance in the community and may not prepare the community to partner in research [6]. Community-Based Participatory Research (CBPR) involves the community in the research process, which helps to develop research questions and contribute to data collection. This process ensures that the research product is relevant and sensitive to the community’s needs [6]. A CBPR approach identities issues defined by the community and attempts to improve local conditions and foster community capacity [7].

The community participated in the training development and contributed actively to providing input on the training content [7]. A CBPR approach was appropriate for confronting some of the misconceptions about COVID-19 and prioritizing community education to counter the misinformation circulating on social media and other unverified information sources [8]. Partnering with FAMU public health researchers in the medically underserved community [9]. also empowered the community by developing and engaging in shaping the research [10]. Community and educational research with community members, trainers, clinicians, and organizations are essential to CBPR [11].

### Study Setting and Overview of Community Engagement Train-the-Trainer Program

The communities in North Florida and selected counties in Central and South Florida provided a train-the-trainer mechanism for COVID-19 vaccine information, outreach, education, and training delivered to primarily African Americans. An extensive train-the-trainer COVID-19 curriculum presentation was a one-and-a-half-hour presentation shaped by the community, trainers, and the FAMU investigators. Faculty experts in behavioral, environmental health, epidemiology, health education, and medicine created it. The trainers and the community reviewed and added content to the presentation. These materials were reviewed and updated continuously. The presentation included cultural humility, disease transmission, health impacts, prevention, the safety of vaccines, facts and myths about vaccines, and vaccine administration. See Table 1 for the outline of the train-the-trainer COVID-19 presentation for train-the-trainers.

### Community Engagement in Research Questions and Materials

A collaborative partnership was encouraged and developed so the community could provide any questions, comments, change concerning the training materials to customize the training to the community needs. The trainer program recruited and trained community trainers who actively trained in the African American community. In addition, the community participated in the data collection of the pre-training and post-training surveys.

The FAMU investigators set up ZOOM meetings with our recruited trainers from a list of trusted trainers in our community.

The COVID-19 curriculum intervention was initially delivered to 20 total trainers from 2021 to 2023. The trainers were provided with a pre-test before the COVID-19 curriculum was delivered, and a post-test to calculate the training’s learning gains and effectiveness. The community trainers included FAMU nursing faculty, Florida Department of Health Trainers, Community Trainers, Area Health Education Centre (AHEC) trainers, community advocate trainers, Community resident input, public health faculty, and students.

The subsequent request from the community trainers was to create a vaccine hesitancy presentation to increase vaccine confidence and acceptance. The trainers suggested a shortened presentation to provide to the community residents. This resulted in a 15-minute presentation, including the fact sheets in English, Spanish, and Haitian Creole with a QR code to access a post survey that focused on vaccine acceptance.

In addition, a 30-minute presentation presented module 6 on facts and myths about vaccines and module 7, vaccine administration (Table 1), followed by the presentation of fact sheets and then a post-survey. The shortened presentation was offered at barber shops, businesses, churches, community events, and small groups. The factsheets on COVID-19 vaccine acceptance in English, Spanish, and Haitian Creole were distributed. Data was collected as a post training but not included in this publication.

Community trainers recruited additional trainers and delivered the training via ZOOM or in person from May 2021 to January 2023. In addition, these community trainers provided additions and feedback to our research team directly from the community, including: 1) reviewing the draft curriculum and providing input regarding community relevancy; 2) pilot assessing and evaluating the curriculum; 3) completing the training modules and pre-and post-surveys; and incorporating the community input. We also conducted an open ZOOM meeting with community trainers to discuss other ways to distribute the training materials effectively. The community trainers attended large public community events where they could distribute fact sheets, answer individual questions from community members, and addressed large crowds.

## Materials and Methods

Two hundred thirty-nine trainers signed the informed consent form to participate in the train-the-trainer initiative. The FAMU Internal Review Board approved the train-the-trainer study [1764181–3], entitled “FAMU COVID-19 Vaccine Community Outreach on June 21, 2021. From July 2021 through January 2023, survey data were collected using Qualtrics©. The initial community trainers were trained virtually. These community trainers recruited by the trained trainers in Florida. There was a pre-survey that collected the knowledge of the trainers before the intervention of training compared to train-the-post-survey included sociodemographic characteristics using the PhenX toolkit questions [12] and included questions taught in the “Stop and Learn Breaks from the PowerPoint training.” In addition, learning gains were calculated.

### Evaluation Methods

A One-Group Pretest-Posttest Design was used to find the learning gains after the intervention training [12]. One hundred forty-six trainers had complete data on the pre- and post-survey answers were included as the population for analysis. Analysis of paired proportions for the community trainers comparing the correct answers before training, the pre-survey, and after the training intervention, the post-survey, quantified the learning gains [13]. The McNemar’s test compared proportions of gains on test scores among participants before and after intervention training. We used the Wilcoxon Signed-Rank test for paired observations to test the hypothesis that there is no difference in the median number of correct answers between the pre- and post-assessment questions [13]. In addition, the community trainer’s satisfaction survey evaluated the COVID-19 training. The Likert scale type questions had answer choices of strongly disagree (1), somewhat disagree (2), neither agree nor disagree (3), somewhat agree (4), and strongly agree (5). Answers scores (mean ± SD; range 1 to 5) with total percentages in complete sentences. The statistical program SAS version 9.4 for Windows (*SAS Institute, Inc., Cary, NC*) was used to analyze the data.

### Results

Ninety-four percent of trainers reported that they had received the COVID-19 vaccine. Eleven percent had previously been ill from COVID-19. Nine percent were concerned about the vaccine side effects, eight percent did not want the vaccine, and two percent did not view COVID-19 as a threat. Most participants were female (84.2%). African American made up 72.6%. with 16.2% white and 15.8% Latino, Asian 4.8% and Native American 1.4%, (Table 2). More than half were employed full-time (53.4%). In addition, the trainer’s educational level was 42.5% postgraduate, with 38%, 26% had Bachelor’s degree, 26% with some college.

The learning gains ranged from 1 (rank 19) to 49 (rank 1). The community trainers collected 146 paired pre-surveys and post-surveys for the COVID-19 train-the-trainer Stop and Learn training. Thirty-two of thirty-five questions showed learning gains (Table 3). Eighteen questions had significant pre-post differences at the α=.05 level. As such, we can conclude that the differences are positive with learning gains, suggesting that there is evidence that the educational intervention improved test scores among the participants (Table 3). Therefore, we can conclude that the differences between pre- and post-learning gains were positive (total +451 learning gains, average learning gain for the group= 8.8%), indicating that the educational intervention improved test scores among the participants (Table 3).

**Figure 1:**
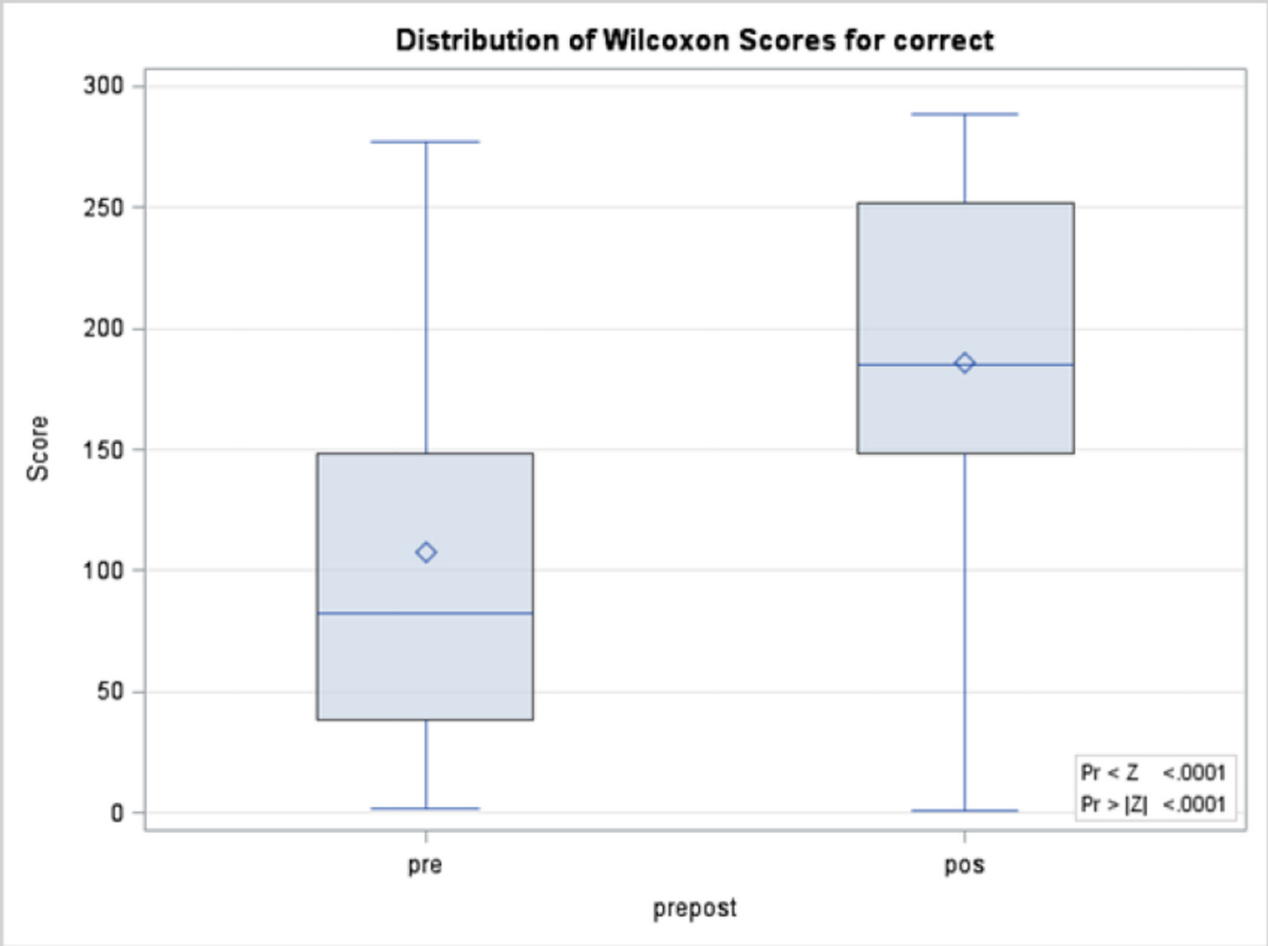
Distribution of Wilcoxon Scores for Correct Answers.

Under the null hypothesis, for a two-sided test, the computed Wilcoxon Signed-Rank test was a Z equal to −7.9793 (p<0.001), indicating statistically significant improved outcomes from the pre-to post-test measurements (Figure 1). The Trainer’s evaluation results are reported in Table 3. The mean answers for the satisfaction for all items were from 4.5 (± 0.9) to 4.8 (± 0.8). The overall satisfaction question had a mean of 4.7 (± 0.7), indicating high satisfaction. The distribution of the total answers was 1.5% for strongly disagree, 2.2% for somewhat disagree, 3.3% for neither agree nor disagree, 12.1% for somewhat agree, and 80.9% for strongly agree (Table 4).

## Implications for Public Health

The community engagement was very timely and helpful to the trainers and the community, and FAMU has strong ties with the local community and community trainers. This longstanding academic-community partnership environment made establishing a network of community trainers easier. These community trainers capitalized on their social capital by looking like the community, representing it, working in it, and sharing values with it. Previously, we had conducted community training with a toxicology curriculum in the community in collaboration with the Duval Health Department which had many partners including Northwest Jacksonville Housing and Neighbourhoods Department, Citizens Planning Committee, East side Environment Council, Jacksonville Community Council, and the Jacksonville Uban League to deliver a toxicology curriculum on a Superfund Site [14]. In addition, we also conducted a previous training for physicians on our toxicology curriculum developed by the Florida Department of Health for physicians, which measured pre- and post-gains [15]. We were able to use these experiences to develop and improve the train-the-trainer project. In the current study, we wanted to increase the number of community trainers. In the future, we would like to incorporate additional referral methods so the community trainers could continue to train other trainers to administer the surveys and continue outreach efforts in other areas of Florida on emerging issues of concern to the community.

### Limitations

The rapid release of COVID-19 information required the need to review updated reports related to COVID-19 and update the presentation continuously and to include community and trainers’ input in updating the training. The COVID-19 Stop and Learn Breaks presentation was continually reviewed for updates every month from May 2021 to September 2022. For example, there were changes in the COVID-19 vaccination recommendations, boosters, and administration intervals.

### Accomplishments of the FAMU Train-the-Trainer

- Conducted the train-the-trainer study during the height of COVID-19
- Approximately seventy-three percent of our trainers were African American
- Approximately 84% were female.
- This initiative trained and assessed 146 trainers with complete information.
- Learning gains comparing Pre- and Post were +451, average learning gain for the group= 8.8%
- Trainers’ evaluations were 4.5 to 4.7 satisfaction out of 5.

## Figures and Tables

**Table 1:** COVID-19 Modules and Topics (Updated 10/2022).

Topic	Process	Materials	Time
Welcome & Introduction	Welcome ParticipantsTrainers Introduction,Cultural Humility	Workshop title [2] Purpose [3] Outline [4]	10 minutes
Module 1-COVID-19 Background	Cause, Origin, disease, and Descriptions of COVID-19. Vaccine and Immunity Herd Immunity	[6–19][16–18] [18–19]	10 minutes
Module 2-Transmission	Transmission, incubation, infection, contact tracing, Self-Quarantine, Self-isolation, face mask type and effectiveness, COVID-19 cases, hospitalizations, and death,	[20–24][25–27][28–29]	10 minutes
Module 3-Health Impacts	COVID-19 symptoms,long term effects, pre exiting conditions,risk factors, Supportive care and testing, risk factors: race/ ethnicity, age and preexisting conditions, boosters, clinical management, treatment, molecular and antibody testing	[33–34] [38–44][45][46–47]	10 minutes
Module 4-Prevention	Prevention, social distancing, hand washing and sanitizing and masking. Routine activities and crowded living conditions. Increase risk of transmission	[51–53]	10 minutes
Module 5-Safety of Vaccine	Safety of vaccines and boosters. mRNA Vaccines, Pfizer-BioNTech and Moderna Johnson & Johnson Janssen vaccines. Update on boosters. Development and safety and common side effects of symptoms of vaccine	[59–63][64–67][68–69][68–70]	10 minutes
Module 6-Facts and myths about vaccines	Misinformation, Things you need to know about vaccines. Overcoming COVID-19 hesitancy, advice for those who are hesitant. Misinformation on vaccines.	74–81	10 minutes
Module 7-Vaccine administration	Why it is important or critical for your safety to get vaccinated. Vaccinations save lives Information on finding sites for vaccination or boosters near you. How to order CO VID-19 testing kits. Provider resources for COVID-19 vaccine conversations with patients.	[82–92]	10 minutes

**Table 2:** Trainers sociodemographic characteristics (n=146).

ChG44:H86racteristics	n (%) or mean ± SD
Age (range 21–73)	37.7 ± 13.3
Female	123 (84.2)
Race [all that apply may >100%]	
White	24 (16.4)
Black/ AA	106 (72.6)
Am Indian/ Alaska	3 (1.4)
Asian	7 (4.8)
Other	7 (4.8)
**Ethnicity**	
No Latino	123 (84.2)
Latino	23 (15.8)
**Education**	
Grades 12/ HS diploma or GED	6 (4.1)
Some college, AD, TD	38 (26.0)
Bachelor’s degree	38 (26.0)
Any postgraduate	62 (42.5)
Refuse to answer	2 (1.4)
**Employment**	
Employed full time	78 (53.4)
Employed part time	16 (11.0)
Self-employed	15 (10.3)
Full time student	24 (16.4)
Part time student	1 (0.7)
Unemployed	6 (2.0)
Unable to work for health reasons	2 (1.4)
Stay at home parent	2 (1.4)
Retired	5 (3.4)
**Income ($ yearly)**	
0 to 9,999	12 (8.2)
10,000 to 14,999	12 (8.2)
15,000 to 24,999	19 (13.0)
25,000 to 34,999	13 (8.9)
35,000 to 49,999	24 (16.4)
50,000 to 74,999	31 (21.3)
≥ 75,000	20 (13.7)
Refuse to answer	15 (10.3)
Where do you live	
House/condo	102 (69.9)
Apartment	40 (27.4)
Mobile home	4 (2.7)
Number of adults	2.1 ± 1.2
Number of children	0.8 ± 1.2
**Marital status**	
Married	46 (31.5)
Widowed	4 (2.7)
Divorced	9 (6.2)
Separated	5 (3.4)
Never married	67 (45.9)
Living together	15 (10.3)
**Medical plan (missing 2)**	
Private	112 (76.7)
Medicare	9 (6.2)
Medicaid	12 (8.2)
No coverage	13 (8.9)

**Table 3:** Pre and post questions (35 items) answered correctly with learning gain difference and rank for each question (n=146).

Questions	Pre- n (%)	Post- n (%)	Post-Pre-Learning Gain difference n (%)	Rank	P-value
A positive COVID-19 test means you may have received the COVID-19 vaccine?	59 (40.4)	108 (73.9)	+49 (33.5)	1	<.0001
Where does COVID-19 rank in terms of cause of death in 2020	52 (35.6)	96 (65.7)	+44 (30.1)	2	<.0001
COVID-19 can be transmitted through using same toilet and bathroom facilities	65 (44.5)	104 (71.2)	+39 (26.7)	3	<.0001
The speed of mRNA manufacturing, government up-front vaccine orders and speed of clinical trials helped speed up vaccine development	89 (61.0)	111 (76.0)	+22 (15.0)	4	0.0054
Messenger RNA (mRNA) vaccines are produced in the laboratory and does not interact with your DNA.	106 (72.6)	127 (87.0)	+21 (14.4)	5	0.0046
Up-front Government ordering vaccine before clinical trials completed for companies to start manufacturing to shorten time for vaccine ready in arms	93 (63.7)	114 (78.1)	+21 (14.4)	5	0.0038
Under what conditions should a person isolate themselves from others?	101 (69.2)	121 (82.9)	+20 (13.7)	6	0.0045
An inactive (dead) adenovirus (cold and flu virus) technology is used in the Johnson & Johnson Janssen COVID-19 vaccine	103 (70.5)	123 (84.2)	+20 (13.5)	6	0.0078
The speed of the clinical trials and emergency review	105 (71.9)	124 (84.9)	+19 (13.0)	7	0.0054
Herd immunity and vaccinations provides immunity to the community	116 (79.4)	132 (90.4)	+16 (11.0)	8	0.0139
Herd immunity takes away my personal freedom	114 (78.1)	130 (89.0)	+16 (10.9)	8	0.0166
African Americans, Hispanics and Native Americans with COVID-19 infections are more likely to be hospitalized and die than white non-Hispanic Whites.	121 (82.9)	137 (93.8)	+16 (10.9)	8	0.0037
African Americans, Hispanics and Native Americans with COVID-19 infections are more likely to be hospitalized and die than white non-Hispanic Whites.	121 (82.9)	137 (93.8)	+16 (10.9)	8	0.0037
The coronavirus is zoonotic, meaning	124 (84.9)	138 (94.5)	+14 (9.6)	9	0.0125
Vaccination builds heard immunity and helps community stays healthy	108 (73.9)	121 (82.9)	+13 (9.0)	10	0.09
A Vaccine immune response helps prevent infection	120 (82.2)	133 (91.1)	+13 (8.9)	10	0.0351
The benefits from the COVID-19 vaccine far outweighs the risk of severe illness and death from COVID-19	130 (89.0)	143 (97.9)	+13 (8.9)	10	0.0044
The COVID-19 shots approved for use in the US are effective and safe.	132 (90.4)	144 (98.6)	+12 (8.2)	11	0.0042
COVID-19 prevents infection for up to 6–8 months	115 (78.8)	126 (86.3)	+11 (7.5)	12	0.12
Immunity can occur through infection from COVID-19	86 (58.9)	96 (65.7)	+10 (6.8)	13	0.28
Transmitting COVID-19 can occur by Coughing, sneezing, talking, or singing loud	132 (90.4)	141 (96.6)	+9 (6.2)	14	0.049
The vaccine builds memory and initiates an immune response quickly	135 (92.5)	142 (97.3)	+7 (4.8)	15	0.11
Have you had COVID-19	121 (82.9)	128 (87.7)	+7 (4.8)	15	0.32
It is important to practice social distancing	139 (95.2)	144 (98.6)	+5 (3.4)	16	0.17
In most cases symptoms related to COVID-19 shots are related to the body’s normal response (immune) to the spike proteins.	136 (93.1)	140 (95.9)	+4 (2.8)	17	0.45
If you are exposed to a person who is positive for COVID-19 and you test negative, you should self-isolate?	97 (66.4)	101 (69.2)	+4 (2.8)	17	0.69
Increase concentration of COVID-19 by infected persons	137 (93.8)	141 (96.6)	+4 (2.8)	17	0.38
A positive COVID-19 antibody test means you may have a strong immune system	107 (73.3)	109 (74.7)	+2 (1.4)	18	0.88
COVID-19 transmission occurs through expelling tiny virus droplets to the air	139 (95.2)	141 (96.6)	+2 (1.4)	18	0.77
It is important to wash hands or use hand sanitizer with at least 60% alcohol	142 (97.3)	144 (98.6)	+2 (1.3)	18	0.68
It is important to wear a mask	142 (97.3)	143 (97.9)	+1 (0.6)	19	1
If you have pre-existing conditions, you should speak with your health care provider before getting the vaccine	143 (97.9)	144 (98.6)	+1 (0.7)	19	1
Persons with COVID-19 may be infectious without symptoms	145 (99.3)	145 (99.3)	0	20	1
Infective droplets from infected persons lungs travel through the air to another person’s lungs, mouth, nose, or eyes resulting in infection	141 (96.6)	140 (95.9)	−1 (0.7)	21	1
COVID-19 is a tiny infectious agent caused by a virus.	142 (97.3)	141 (96.6)	−1 (0.7)	21	1
Total gain difference and average %	+451 (8.8)				

**Table 4:** Trainers Evaluations, N=25.

Question	Strongly disagree	Somewhat disagree	Neither agree nor disagree	Somewhat agree.	Strongly agree.	Mean ± SD
The community train-the-trainer curriculum is useful tool for providing information to the community (n=25).	2			2	21	4.6 ± 1.1
The materials I received were useful for guiding me through the training session (n=25).	1		1	3	20	4.6 ± 0.9
The training was well organized, and time was used efficiently (n=25).		1	1	2	21	4.7 ± 0.7
The facilitator used clear, simple language that I could understand (n=25).			1	4	20	4.8 ± 0.5
The training included a clear explanation of what was expected of me as a community trainer (n=24).		1	1	2	20	4.7 ± 0.8
The length of the training was appropriate for material that was presented (n=25).		2	1	5	17	4.5 ± 0.9
There was time for questions and my comments received feedback from the facilitator (n=25).			2	4	19	4.7 ± 0.6
There was enough variety of content to keep me interested (n=23).		1	1	1	20	4.7 ± 0.8
The facilitators that trained me were knowledgeable and able to effectively explain essential information (n=25).	1			2	22	4.8 ± 0.8
The training was delivered in a culturally sensitive manner (n=25).				5	20	4.8 ± 0.4
Overall, I feel satisfied with the training and materials provided (n=25).		1	1	3	20	4.7 ± 0.7
Total answers n (%)	4 (1.5%)	6 (2.2%)	9 (3.3%)	33 (12.1%)	220 (80.9%)	272 (100%)
